# Economic evaluation of diverting low-risk patients from the emergency department to adjacent out-of-hours primary care in Belgium

**DOI:** 10.1017/S146342362610142X

**Published:** 2026-07-30

**Authors:** Jane Phiri, Stefan Morreel, Diana De Graeve, Philippe Beutels, Anthony Pairon, Hilde Philips, Koen Monsieurs, Veronique Verhoeven, Lander Willem

**Affiliations:** 1 Department of ELIZA (Primary and Interdisciplinary Care), Universiteit Antwerpen, Antwerp, Belgium; 2 Department of Economics, Universiteit Antwerpen, Antwerp, Belgium; 3 Centre for Health Economic Research and Modelling Infectious Diseases, Universiteit Antwerpen, Antwerp, Belgium; 4 Emergency Department, Antwerp University Hospital, Universiteit Antwerpen, Antwerp, Belgium

**Keywords:** economic evaluation, emergency department, healthcare savings, hospitalization, out-of-hours primary care, time spent at the facility, triage

## Abstract

**Background:**

Out-of-hours primary care (OOH-PC) has been introduced in many health systems to reduce non-urgent emergency department (ED) use. While prior studies suggest potential efficiency gains, economic evaluations rarely stratify outcomes by patient risk, limiting policy relevance. This study assesses the cost-effectiveness of diverting low-risk patients from ED to OOH-PC.

**Aim:**

Compare direct medical costs, time spent at the facility, and hospitalization probability across risk and care setting subgroups from a societal perspective.

**Methods:**

We analysed data from a cluster-randomized trial conducted in Belgium that examined a nurse-led triage system. Under the study trial’s extended Manchester Triage System (eMTS) protocol, patients eligible for primary care were assigned to OOH-PC and are referred to in our study as low-risk, while those assigned to the ED are defined as high-risk. To complement previous work, we stratified costs and effects by risk group. Mixed-effects regression models estimated costs, time at the facility, and hospitalization, with bootstrap confidence intervals. We also calculated the incremental net monetary benefit (INMB).

**Results:**

Treating low-risk patients at the OOH-PC instead of at the ED reduced costs by €24 (€14–€39) per patient, shortened the time spent at the facility by 69 (52–94) minutes. From a societal perspective, the average INMB was €49.5 per low-risk patient. Aggregately, we estimated that the 37 intervention weekends in 2019 led to an average saving of €14,136 in direct medical costs and cumulative time savings of 28 days.

**Conclusion:**

Diverting low-risk patients from the ED to OOH-PC resulted in cost- and time-savings for low-risk patients at the study site. These findings highlight the potential to improve out-of-hours healthcare delivery to maximize benefits for patients and healthcare systems, although confirmation in other healthcare settings is required before wider implementation.

## Introduction

In the face of rising healthcare needs and expenditures, there is a growing emphasis on enhancing the efficiency of the healthcare system. Emergency departments (EDs) play a crucial role in providing out-of-hours care but overcrowding threatens the timeliness and quality of care (Francetic *et al*., 2024). There is a growing scope for EDs to divert low-risk patients to more appropriate care settings. Out-of-hours primary care (OOH-PC) services, alongside EDs or standalone, have gained recognition as alternative care settings for low-risk patients (Berchet, 2015; Broekman *et al*., 2017; Cooper *et al*., 2019; Schoenmakers *et al*., 2021). OOH-PCs are generally defined as primary care services operational from 6:30 pm to 8:00 am on weekdays and all day on weekends and public holidays (Berchet, 2015). OOH-PC can ensure appropriate and timely access to primary care services outside regular GP hours (Berchet, 2015). This strategy can improve patient satisfaction, reduce waiting time, and decrease hospitalizations through timely access to care (Berchet, 2015; Møller *et al*., 2018).

Despite the potential benefits of OOH-PC, there is a paucity of comprehensive economic evaluations in the literature to critically appraise its value to healthcare systems and society (Phiri *et al*., 2024). While previous studies have assessed OOH-PC as an alternative to ED care, they have focused on specific subpopulations, such as alcohol-intoxicated patients (Moore *et al*., 2021) and pediatric patients (Poole *et al*., 1993; Sterner *et al*., 2012; Flaherty *et al*., 2022), which makes it difficult to generalize the findings to all patients seeking out of hours and/or ED care. Furthermore, the potential impact of OOH-PC on patients with different risk levels in different treatment settings (i.e., at the ED or at the OOH-PC) requires differentiated results to enable tailored decision-making. Overlooking risk heterogeneity may lead to biased conclusions, as low- and high-risk patients typically follow distinct care pathways and incur different costs. Economic evaluations that explicitly stratify outcomes by risk level remain scarce. To date, few studies have systematically compared OOH-PC and ED care using risk-based stratification, suggesting an important gap in the evidence base for healthcare planners aiming to optimize resource allocation and efficiency. In Belgium, research considering the economic aspects of OOH-PC has been conducted. Notably, in 2019, a cluster randomized controlled trial (RCT) called the ‘TRIAGE trial’ was conducted at the ED of an urban hospital in Belgium and its adjacent OOH-PC (Morreel *et al*., 2022a). Previous work on the TRIAGE trial included a process evaluation and concluded that the new tool safely diverted 10% of the included patients to primary care. Furthermore, hospitalization probabilities were estimated and comparisons in direct medical costs were made from patient, provider, and insurance perspectives (Homburg *et al*., 2022; Morreel *et al*., 2022a; 2022b). However, previous analyses did not focus yet on patient groups based on both risk level and setting (either OOH-PC or ED).

In this secondary analysis of a cluster randomized trial, we aim to evaluate the cost-effectiveness of diverting low-risk patients from the ED of an urban hospital in Belgium to an adjacent OOH-PC facility. In the current analysis, we stratify costs and effects by risk levels and settings, to account for differences in the ratio of low- and high-risk patients over time, as these have different attributable costs.

We hypothesize that treating low-risk patients at OOH-PC is more cost effective than treating them at the ED, with time loss and hospital admissions as effect measures. Additionally, we postulate that hospital admissions at the OOH-PC could be lower compared to the ED, when risk level is held constant. Based on reduced crowding, we postulate that treating the remaining low-risk patients in the ED is more cost-effective during periods when other low-risk patients are diverted to OOH-PC, compared to periods without such a diversion.

## Methods

### Setting and study design

The study was conducted in Belgium, where OOH-PC is organized through general practitioner cooperatives (GPCs) and are either integrated with EDs to streamline patient flow or operate independently (Schoenmakers *et al*., 2021). A brief description of the Belgian healthcare system and financing context is provided in Appendix A.

This study builds upon a previously conducted cluster randomized control trial - the TRIAGE trial – to assess an extended Manchester Triage System (eMTS) in an urban ED and the adjacent OOH-PC (Morreel *et al*., 2022a) in Belgium. In this study, the MTS (v3.6) was integrated into a computer decision support system (E.care ED 4.1) and extended with the assignment to either OOH-PC or ED (Morreel *et al*., 2022b). Although the OOH-PC in this study is located adjacent to the ED, it operates as an independent GP-led primary care service, with separate clinical governance, staffing, and tariff structures from the hospital. Its location is organizational rather than clinical, and care delivery remains consistent with community-based primary care practice.

Weekends (Friday 7 p.m. until Monday 6 a.m.) or bank holidays, hereinafter referred to as weekends, were the units of randomization, and patients were the units of analysis (Morreel *et al*., 2022a). Starting from the 1^st^ of March 2019 until the 31^st^ of December 2019, for every three intervention weekends, the fourth weekend was designated as a control weekend. Ultimately, there were 37 intervention weekends and 10 control weekends.

During intervention weekends, nurses assigned an urgency score using eMTS to patients and advised low urgency patients eligible for primary care to seek care at the OOH-PC next door, and other patients to remain at the ED (Morreel *et al*., 2022a). On control weekends, nurses also assigned patients an urgency score with eMTS, but only virtually assigned them to the ED or OOH-PC. Patients remained at the ED since nurses did not communicate patients’ assignment on control weekends (Morreel *et al*., 2022a).

Outside the trial setup, referral from an ED to OOH-PC is uncommon, since staff typically respect patients’ decision to present to the ED. On intervention weekends, nurses communicated the eMTS assignment advice to patients; but they did not on control weekends. Clinical practice during both control and intervention weekends was identical. When assuming patients made decisions independently and did not systematically exchange information regarding waiting times or costs across weekends, we are convinced the study design shows minimal spillover effects in patients decision-making between ED and OOH-PC. We obtained pseudonymized patient-level data via iCAREdata, a database of out-of-hours care encompassing data from the EDs and GPCs, including health care utilization, socio-demographic information, time stamps for triage and diagnosis, and hospitalization (Colliers *et al*., 2016).

### Risk classification and analysis groups

**Figure 1.**
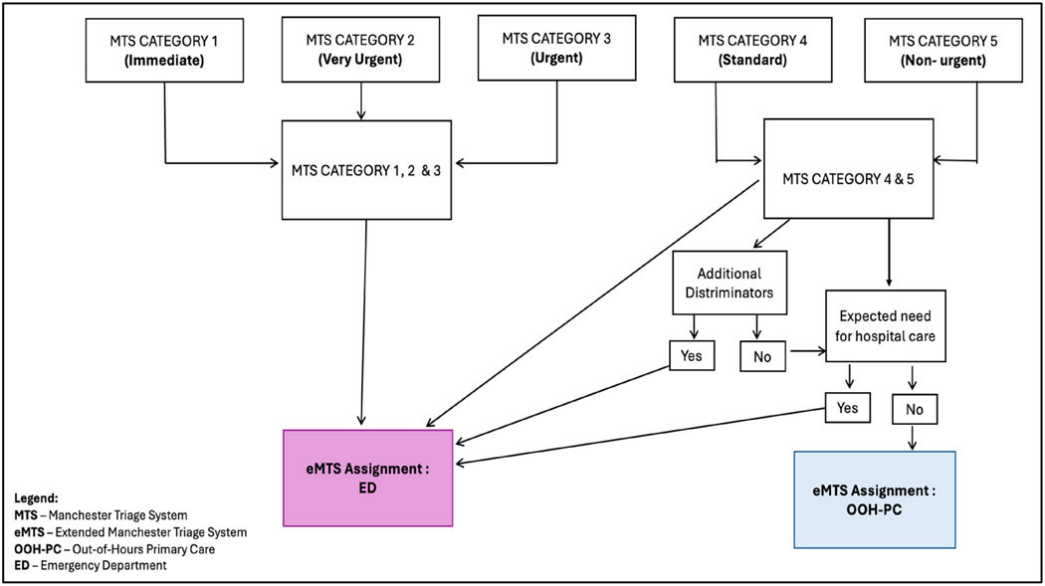
Figure 1 long description. Triage flow of patients leading to ED or OOH-PC assignment.

In the TRIAGE study, triage started from a presentation-specific flow chart (e.g., chest pain) and worked through predefined ‘discriminators’ to assign an urgency category (1–5; 1 – Immediate, 2 – Very Urgent, 3 – Urgent, 4 – Standard, and 5 – Non-urgent). By applying the eMTS, categories 1 to 3 always resulted in an assignment to the ED. Categories 4 and 5 could lead to either assignment to the OOH-PC or ED based on supplementary discriminators. All patients aged under three months were always assigned to the ED, regardless of their triage category. For each OOH-PC assignment, triage nurses could override this decision based on a patient’s expected need for hospital care (radiology, complex interventions, hospitalization), which resulted in an assignment to the ED (Morreel *et al*., 2022b). See Figure 1 for an illustration of the triage flow. Further details of classification have been described by Morreel *et al*. (2022b). In this study, we therefore define low-risk patients as those assigned to the OOH-PC and high-risk patients as those assigned to the ED. Figure 1 illustrates the triage flow leading to ED or OOH-PC assignment. This design enabled the creation of five analysis groups. During intervention weekends, three groups were created:

1. Low-risk patients at OOH-PC during the intervention: patients assigned to the OOH-PC and handled at the OOH-PC during intervention weekends.
2. Low-risk patients at the ED during the intervention: patients assigned to the OOH-PC and handled at the ED during intervention weekends.
3. High-risk patients at the ED during the intervention: patients assigned to the ED and handled at the ED during intervention weekends.

During control weekends, two groups were created:

1. Low-risk patients at the ED during the control: patients virtually assigned to the OOH-PC and handled at the ED during control weekends.
2. High-risk patients at the ED during the control: patients virtually assigned to the ED and handled at the ED during control weekends.

**Figure 2.**
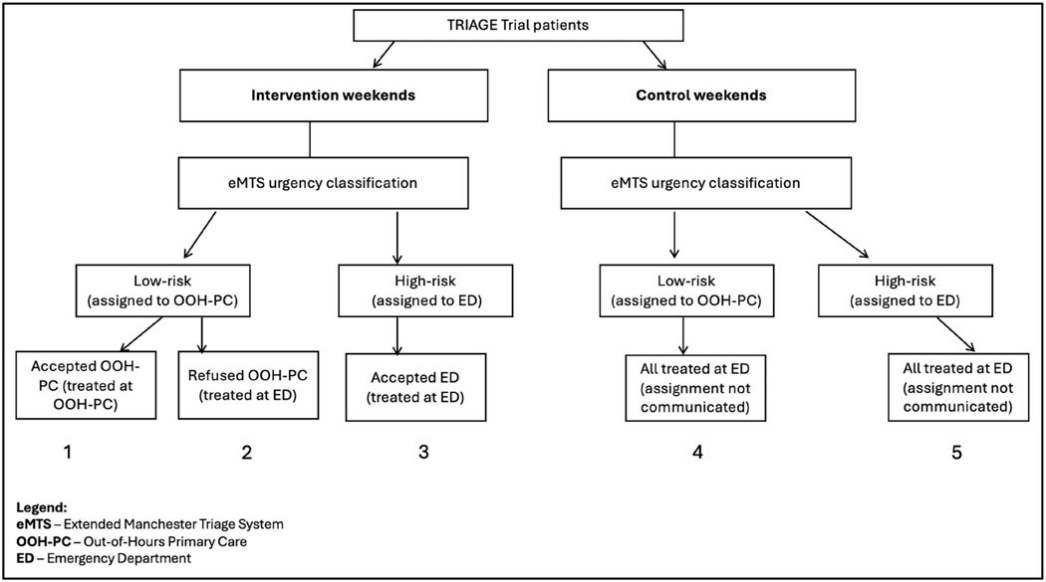
Flow of patients from triage to analysis groups.

Figure 2 presents the flow of patients from triage to the analysis groups.

### Cost estimation

We conducted our analysis from the healthcare payer perspective by aggregating the expenses incurred by the patient and the health insurance based on medical nomenclatures and invoice data collected from the billing departments of the hospital and OOH-PC. Morreel *et al*. (2022a) details the process of translating nomenclatures into costs and matching invoice data with medical records based on sex, birth year, postal code, and time. Table 1 provides an overview of the cost categories included.

**Table 1.** Cost categories and descriptions Table 1 long description.

Cost category	Description
Ambulatory physician fees	-
Medical imaging	E.g. radiology procedures
Technical procedures	E.g. sutures, plastering
Medications	Medication costs only cover the medication administered to the patient during a consultation, excluding any prescriptions provided
Non-refundable items	The non-refundable items category includes various articles requested by the patient (e.g., a toothbrush) or necessary for their medical care (e.g., crutches) (Morreel *et al*., 2022a).
Supplemental fees	Night-time and weekend consultation surcharges

The primary analysis of the Triage Trial excluded patients who were subsequently hospitalized (Homburg *et al*., 2022; Morreel *et al*., 2022a). In contrast, our study includes these patients. However, note that the costs for hospitalized patients do not represent the full costs of inpatient admission. Additionally, certain combinations of nomenclature codes performed in the ED and during subsequent inpatient care cannot be billed simultaneously. As a result, some procedures initiated in the ED may not be billed, which could lead to some minor underestimation of ED costs. This approach allows us to provide an overview of the financial implications associated with ambulatory patient care for both hospitalized and non-hospitalized patients. Data capturing did not include broader cost categories such as indirect medical costs or patient transportation costs. We did not consider long-term costs or effects; therefore, we did not apply discounting.

### Effect estimation

We evaluated effectiveness using two measures: time spent at the facility and hospitalization probability. The trial staff captured hospital admission as either ‘yes’ or ‘no’ as part of the trial visit, but data on admissions at subsequent visits were not available. In Belgium, hospital admission is solely a medical decision made by the ED physician and is not subject to approval of health insurers; reimbursement follows standard national tariffs, and this process applies equally to patients presenting via ED or referred from OOH-PC. We approximated the time spent at the facility by calculating the number of minutes from the time stamp of the initial ED triage to the time stamp of the physician’s diagnosis at the care facility. This time difference serves as a proxy for the duration patients spend at the facility. To improve the validity of the proxy measure, we excluded records with likely technical or administrative issues, indicated by times differences less than zero or exceeding 480 minutes. We chose 480 minutes as a cut-off, as it represented the 95^th^ percentile, after the exclusion of negative values of the time spent at the facility distribution (Weichle *et al*., 2013).

### Regression analysis

We conducted a model-building process to identify the optimal regression models for the cost and effects. Potential covariates included setting (OOH-PC or ED), intervention (Intervention or Control), risk level (low-risk or high-risk), sex (Male or Female), clinician ID (24 unique clinicians), and interaction effects between risk level, setting and intervention arm. We evaluated all covariates for their contributions to linear regression, logistic regression, and mixed-effects models. This evaluation utilized both forward and backward elimination processes. We selected the best-fitting model based on the Akaike Information Criterion (AIC), which allowed us to choose the model ensuring a balance between model fit and complexity. After the model selection, we used the best-fitting model to estimate costs and effects for the five patient groups.

For the cost analyses, the best model was a linear mixed-effects regression, with cost as the dependent variable, incorporating a three-way interaction between risk level, setting, and intervention, with age and sex as covariates and clinician as a random effect.

For the time spent at the facility analysis, the best-fitting model was a linear mixed-effects regression model that included a three-way interaction between risk level, setting, and intervention, along with age and sex as covariates and clinician as a random effect.

For the probability of hospitalization, we evaluated all covariates for their contributions to both logistic regression and generalized linear mixed-effects models. The best-performing model, in this case, was a generalized linear mixed-effects model, which included the three-way interaction of risk level, setting, and intervention, along with age and clinician as random effects.

For all outcomes, we estimated mean values based on 8,000 bootstrap replications. To assess uncertainty surrounding these estimates, we computed bias-corrected and accelerated (BCa) 95% confidence intervals. We selected BCa intervals because of their robustness in handling non-normal and skewed distributions (Wicklin, 2017), which was observed in the cost distribution. Full details of model building are provided in Appendix B.

### Cost-effectiveness analysis

We conducted two sets of comparative economic evaluations focusing on the two effects: time spent at the facility and hospitalization. We conducted four comparisons between different patient groups:

- Low-risk patients treated at OOH-PC during the intervention versus low-risk patients treated at the ED during the intervention.
- Low-risk patients treated at OOH-PC during the intervention versus low-risk patients treated at the ED during the control.
- Low-risk patients treated at the ED during the intervention versus low-risk patients treated at the ED during the control.
- High-risk patients treated at the ED during the intervention versus high-risk patients treated at the ED during the control.

In both the time spent at the facility and hospitalization analyses, we estimated incremental costs and effects for each pairwise comparison. As done for the individual patient group estimates, we assess the uncertainty surrounding these estimates, by computing BCa confidence intervals based on 8,000 replications. We also estimated a point estimate of the incremental cost-effectiveness ratio (ICER) for each pairwise comparison using the ratio of means approach as recommended by Stinnett et al., dividing the mean incremental cost by the mean incremental effect for 8000 bootstrap samples (Stinnett and Paltiel, 1997). Therefore, ICERs are not reported with confidence intervals.

We estimated the incremental net monetary benefit (INMB) for the time spent at the facility analysis using a willingness-to-pay value of €0.3616, which was the average wage per minute based on the national average monthly wage of €3758 for Belgium in 2019 (STATBEL, 2021), and assuming a 40-hour work week. The NMB is a summary measure that expresses the value of an intervention in monetary terms when a willingness-to-pay threshold for one unit of benefit is known, and is calculated as [(incremental effect × willingness-to-pay threshold) − incremental cost] (York Health Economics Consortium 2025). The INMB represents the difference in NMB between two strategies; a positive INMB indicates that the intervention is cost-effective at the specified willingness-to-pay threshold (York Health Economics Consortium 2025). For hospitalization, we were unable to estimate INMB due to the absence of a reference estimate reflecting willingness to pay to avoid a 1% increase in hospitalization probability. Additionally, we conducted a probabilistic sensitivity analysis (PSA) to assess the uncertainty associated with the estimated INMB values, calculated using the willingness-to-pay threshold of €0.3616 per minute. We performed this by estimating the probability of the INMB values being positive through 10,000 simulations. The distribution of both costs and time spent at the facility fits a gamma distribution, which served as the basis for the PSA.

Furthermore, as a sensitivity analysis, we applied a −75% to +200% variation to willingness-to-pay threshold to calculate INMB values for the same pairwise comparisons in the time spent at the facility analyses. Using this same −75% to +200% variation, we conducted a PSA to estimate the percentage of times the INMB values are positive.

### Total costs and effects

Total cost and effect estimations for the patient population at the study ED started from the obtained incremental costs and effects of treating low-risk patients at OOH-PC compared to treating them at the ED during the intervention weekends. We multiplied the estimated costs by the reported number of low-risk patients that presented themselves at the study ED. To estimate the potential annual impact, the patient population of the 37 intervention weekends from 1^st^ March 2019 to 31^st^ December 2019 was scaled to 52 weekends to obtain an annual estimate. We did not extrapolate observed changes in hospitalization probability to an annual rate or across facilities, because we do not expect probability changes to be additive.

### Data analysis

All analyses were conducted in R (version 4.4.0; 2024-04-24). Full details of software versions and packages used are provided in Appendix C.

## Results

### Setting and population

The TRIAGE trial dataset comprised 8158 records of patients who presented at the ED during the study period. To create our analysis datasets, we excluded 282 records for patients who left without being seen, as they were missing all three values: costs, time spent at the facility, and clinician (doctor) categorization of urgency during the consultation. Additionally, we excluded 89 records missing a risk allocation at triage and information about the care setting in the intervention arm. Furthermore, we excluded 95 records where both an OOH-PC and ED time stamps were registered. We excluded 65 records missing clinician identity, to make sure we can account for between-clinician differences in patient administration. To analyse costs, we further excluded 173 records missing cost and we ultimately had 7454 records. For the time spent at the facility analysis, 3099 records were missing a time of diagnosis and 115 had an estimated time spent at the facility less than 0 minutes or exceeding 480 minutes, resulting in 4240 records for the time spent at the facility analysis. For the hospitalization analysis, we used the subset of 7454 records that we also used for the cost analysis, as there were no additional missing data issues for this variable. The stepwise creation of the analytical datasets is illustrated in Appendix D.

### Costs and effects

During the intervention weekends, low-risk patients that agreed to consult OOH-PC had on a mean cost of €57.7, a mean time spent at the facility of 34.1 minutes and had a hospitalization probability of 0.009. In contrast low-risk patients that opted to stay in the ED had on average a higher cost (€81.7), spent more time at the facility (103.3 minutes), and had a higher hospitalization probability (0.059). High-risk ED patients during the intervention had the highest mean costs (€129.6), a mean time spent at the facility of 104.8 minutes, and a mean hospitalization probability of 0.205 (Table 2).

**Table 2.** Number of weekends, patient population sizes, medical costs, time spent at the facility, and hospitalization probability in terms of the mean and bias-corrected and accelerated 95% confidence intervals Table 2 long description.

Population	Number of weekends	Population size (cost and hosp. analysis)	Population size (time analysis)	Medical cost (€)	Time spent at care facility (minutes)	Hospitalization probability
Low-risk patients at OOH-PC during the intervention	37	589	585	57.7 (53.2, 62.1)	34.1 (19.8, 49.0)	0.009 (0.002, 0.050)
Low-risk patients at ED during the intervention	37	172	87	81.7 (73.1, 94.7)	103.3 (90.4, 122.4)	0.059 (0.027, 0.115)
High-risk patients at ED during the intervention	37	5070	2723	129.6 (126.1, 134.1)	104.8 (98.8, 112.5)	0.205 (0.186, 0.237)
Low-risk patients at ED during the control	10	401	213	81.3 (75.2, 89.9)	97.2 (88.7, 110.4)	0.062 (0.039, 0.101)
High-risk patients at ED during the control	10	1222	632	128.8 (122.2, 137.1)	101.9 (94.7, 111.1)	0.237 (0.207, 0.284)

ED = emergency department; OOH-PC = out-of-hours primary care.

For the control weekends, low-risk ED patients had a mean cost of €81.3, a mean time spent at the facility of 97.2 minutes, and a mean hospitalization probability of 0.062, while high-risk ED patients had a mean cost of €128.8, a mean time spent at the facility of 101.9 minutes, and a mean hospitalization probability of 0.237 (Table 2).

### Cost-effectiveness analyses

#### Comparison A and B: Low-risk patients treated at OOH-PC during the intervention versus low-risk patients treated at the ED (intervention and control periods)

Treating low-risk patients at the OOH-PC instead of the ED was cost- and time-saving and reduced the hospitalization probability (Table 3). Specifically, low-risk patients at OOH-PC incurred €24 lower costs on average, spent 69 fewer minutes at the facility, and had a 0.049 lower probability of hospitalization compared to low-risk patients who chose to remain at the ED during the intervention period.

**Table 3.** Cost-effectiveness results for time spent at the facility and hospitalization when comparing different patient populations, as described in the methods section (comparison A–D) Table 3 long description.

Comparisons	Incremental cost (BCa 95% CI)	Time spent at the facility				Hospitalization	
		Reduced time at facility (BCa 95% CI) (min)	ICER – cost per reduction in time spent at the facility (€/min) (€/hour)	INMB (time spent at the facility (95% CI)	INMB – % positive	Reduced Hospitalization probability (BCa 95% CI)	ICER – Hospitalization probability
(A) Low-risk patients at OOH-PC during the intervention vs low-risk patients at the ED during the intervention	−€24.0 (−39.3, −14.3)	69.3 (51.6, 93.5)	−€0.35/min −€20.78/hour	€49.5 (9.3, 77.3)	85.5%	0.049 (0.011, 0.106)	−492.1
(B) Low-risk patients at OOH-PC during the intervention vs low-risk patients at ED during the control	−€23.6 (−34.1, −16.3)	63.1 (48.8, 82.6)	−€0.37/min −€22.39/hour	€46.7 (22.3, 71.1)	84.6%	0.052 (0.023, 0.090)	−452.9
(C) Low-risk patients at ED during the intervention vs low-risk patients at ED during the control	0.4 (−10.9, 13.7)	−6.1 (−24.6, 10.1)	−€0.07/min −€4.12/hour	−€2.8 (−23.6, 18.0)	46.7%	0.003 (−0.048, 0.048)	128.3
(D) High-risk patients at ED during the intervention vs high-risk patients at ED during the control	0.8 (−7.7, 8.2)	−2.8 (−8.1, 3.6)	−€0.28/min −€16.67/hour	−€1.7 (−9.1, 5.6)	57.7%	0.033 (0.001, 0.066)	23.8

BCa = Bias corrected and accelerated; CI = confidence Interval; ICER = incremental cost-effectiveness ratio; INMB = incremental net monetary benefit; min = minutes; €/min = Euros per minute; ED = emergency department; OOH-PC = out-of-hours primary care.

Similar results were observed comparing low-risk patients at OOH-PC to low-risk patients treated in the ED during the control weekends. In both comparisons, the BCa 95% CI for the incremental costs and effects did not include zero. The INMB values based on time spent at the facility were positive when applying the average wage in Belgium of €0.3616 per minute as willingness-to-pay threshold. Our PSA showed that the INMB values for time spent at the facility were positive in at least 84% of the simulations. Further sensitivity analysis showed that the INMB for time spent at the facility remains on average above zero when applying a 75% lower willingness-to-pay threshold. More details on PSA and the sensitivity analysis are available in Appendix E.

#### Comparison C: Low-risk patients treated at the ED during the intervention versus low-risk patients treated at the ED during the control

We had inconclusive results when comparing low-risk patients at ED during the intervention with low-risk patients at ED during the control. For these comparisons, mean costs, time spent at the facility, and hospitalization probability were not significant since the CI included zero.

#### Comparison D: High-risk patients treated at the ED during the intervention versus high-risk patients treated at the ED during the control

Likewise, we had inconclusive results when comparing high-risk patients at ED during the intervention with high-risk patients at ED during the control. However, an exception was the difference in hospitalization probability when comparing high-risk patients at the ED during the intervention period with those during the control period. However, due to the insignificant cost differences, the overall cost-effectiveness remains inconclusive.

### Total cost and effect

Our analysis focusing on low-risk patients showed that treating them at OOH-PC during weekends instead of the ED resulted in average savings of €14,136 aggregately, for the 37 intervention weekends. This could be translated into an annual aggregate cost saving of about €19,867 for this study site. The expected annual savings for all low-risk patients presenting at the study ED, including those who did not accept the allocation decision, would amount to approximately €25,668 for the study site. During control weekends, nurses triaged a higher proportion of patients as low-risk (Morreel *et al*., 2022b), which would increase the potential annual savings for this ED to €50,045. This calculation assumes that the care provided to these patients at the OOH-PC is equivalent to the care they would receive at ED. All costs are included from the healthcare payer perspective, thus accounting for costs borne by both patients and health insurance.

Treating low-risk patients at OOH-PC instead of the ED reduced the total time spent at the care facility by 28 days for all included patients over 37 study weekends, translating to approximately 40 days annually for the study site. When including low-risk patients who declined referral to the OOH-PC, annual time savings for patients could amount to 51 days for the study site. When extrapolating the reported number of low-risk patients from control weekends, annual time savings for all patients could sum up to 100 days for the study site, if all low-risk patients are treated at OOH-PC.

## Discussion

In this secondary analysis of the TRIAGE trial, we analysed and compared the costs, time spent at the facility, and probability of hospitalization across various subpopulations by risk and setting. Our study demonstrated significantly lower costs, shorter time spent at the facility, and reduced hospitalization probability for low-risk patients when referred from the ED to OOH-PC. These findings hold when OOH-PC is compared with low-risk patients in the ED during the intervention and control weekends. This could be based on the premise that patients at GPs typically experience shorter wait times compared to EDs, where prioritization based on risk level can delay care for low-risk patients (Elkum *et al*., 2009). In an ED, high-risk patients require immediate attention, leading to delays in seeing lower-risk patients. In contrast, OOH-PCs primarily handle low-risk patients, thereby eliminating competition with high-risk patients.

Our results align with a prior study in the United States of America, which found that low-risk pediatric care at GPs was less costly and had a shorter length of stay than care at the ED (Sterner *et al*., 2012). Interestingly, another American study found that appointment-based family practice centers, despite lower costs, had longer wait times from registration to seeing a clinician compared to free-standing EDs (Chesteen *et al*., 1986). Unlike our study, these study results are likely influenced by the difference between appointment-based and walk-in healthcare services. It is important to note, that the healthcare system in the United States differs considerably from that of Belgium, limiting direct comparison, but still providing perspective. In general, long wait times pose several public health challenges, including reduced access to care, disruptions in hospital workflows, and impacts on the efficiency and on the quality of health services (Walle *et al*., 2024).

Regarding hospitalization of low-risk patients, we observed significant differences by setting. Specifically, low-risk patients treated at the OOH-PC had a lower hospital admission rate of 1% compared to 6% for low-risk patients treated at the ED, both during intervention and control weekends. Note that previous studies using MTS found hospitalization rates ranging from 1% to 2% for low-risk patients (Roukema *et al*., 2006; Martins *et al*., 2009; Van Der Wulp *et al*., 2009). The variations in hospitalization rates across studies may be attributed to differences in study design, patient populations, healthcare settings, highlighting the importance of context-specific evaluations. In this current study, lower hospitalization probability for low-risk patients at OOH-PC may reflect differences in clinical decision-making between general practitioners and emergency physicians. Previous studies suggest that GPs may, in some settings, manage diagnostic uncertainty using a ‘wait and see’ approach, whereas ED practices may tend to favor immediate investigation and lower risk tolerance, partly due to medico-legal pressures and high-acuity context (Gaughan *et al*., 2022). Prior work also shows that primary care services use fewer diagnostic resources and adopt more conservative admission thresholds for low-risk conditions (Berchet, 2015; Smits *et al*., 2019). Together, these factors may partly explain observed results in our study. Future mixed-methods research could explore how differences in clinical culture and risk perception shape triage and treatment decisions.

Previous secondary analyses of the TRIAGE trial revealed that nurses classified a relatively higher number of patients as high-risk during intervention weekends, resulting in proportionally more referrals to the ED (Morreel *et al*., 2022b). The primary reasons for this increased high-risk classification during intervention visits were largely based on nurses’ perceived necessity for medical imaging or other medical considerations not specified in the protocol (Morreel *et al*., 2022b). This difference in case mix suggests that the possible higher proportion of complex or borderline high-risk patients classified as low-risk during the control period potentially inflated time, cost and hospitalization estimates in the comparison of low-risk at OOH-PC during the intervention versus low-risk patients at the ED during the control. However, our findings remain consistent when comparing low-risk patients treated at the OOH-PC firstly with low-risk patients treated at the ED during the intervention (i.e. refused triage advice), and secondly with low-risk patients treated at ED during the control (when no triage advice was given). This consistency could indicate that our results in the latter case are not significantly affected by the differences in nurses’ risk level classifications across the intervention and control periods.

The estimated INMB was €49.5 per low-risk patient treated at the OOH-PC compared with low-risk patients treated at the ED during intervention weekends, and €46.7 when compared with low-risk patients treated at the ED during control weekends. In practical terms, this means that after accounting for both the lower costs and the monetized value of substantially shorter time spent at the facility redirecting a low-risk patient to the OOH-PC generates about €50 more value for the healthcare system than managing the same patient at the ED, during the intervention or control periods.

It is important to note that the magnitude of the INMB is inherently dependent on the selected willingness-to-pay threshold, which can introduce a systematic source of bias. Because the Net Monetary Benefit framework converts effects into monetary terms using the chosen willingness-to-pay value, different thresholds would produce different INMB estimates even when the underlying cost and time differences remain unchanged. In our analysis, the approximately €50 INMB reflects the net gain conditional on valuing each minute saved at €0.3616; a higher willingness-to-pay threshold would increase the INMB, while a lower threshold would reduce it.

When focusing on high-risk patients, we observed on average, fewer hospital admissions during intervention weekends compared to the control weekends. Low-risk visits to the ED can strain resources, including healthcare professionals’ time and medical equipment, diverting them from attending to high-risk patients (Berchet, 2015). Consequently, a high volume of low-risk patients in the ED can delay diagnoses or treatments of higher-risk patients (Berchet, 2015; Durand *et al*., 2012) and increase hospital admissions and readmissions (Ben-Yakov *et al*., 2015). Note that these findings reflect associative effects, and we are not able to account for or compare differences in case mix. Specifically, we observed that the probability of hospitalization for high-risk patients at the ED was 21% during the intervention period and 24% during the control period. This may relate to the observation by Morreel et.al. that triage tended to be more conservative when the intervention was active (Morreel *et al*., 2022b). However, it remains notable that we do not see a comparable shift among low-risk patients presenting at the ED across either intervention arm. The presence of overlapping confidence intervals does not necessarily imply the absence of a significant incremental difference (Goldstein and Healy, 1995). This phenomenon is evident in our findings, where despite overlap in the confidence intervals of individual hospitalization rates for high-risk patients at the ED during the intervention and control periods, the incremental differences were statistically significant.

Our findings indicate economic and operational advantages for low-risk patients when diverted from ED to OOH-PC facilities. For the study ED and its co-located OOH-PC patient population, the potential cost savings from the healthcare payer perspectives ranged from €20,000 to €50,000 annually. In addition, this implies potential total time savings of up to 100 days per year for this patient group, allowing them to make the most of their personal and leisure time after hours and on weekends, enhancing their overall well-being and quality of life. These estimations do not account for practical or organizational restrictions. It is important to note that the lower bound likely underestimates the full potential for diversion, as it reflects conservative assignment practices during the intervention. In contrast, the upper bound may overestimate this potential, as it does not account for patients’ preferences in adhering to the triage recommendation. As such, the potential costs and time benefits depend on the willingness of patients to accept triage decisions, which highlights the importance of improving public awareness and trust in the triage and OOH-PC services (Zhang *et al*., 2025). In this regard, further research is necessary to comprehensively assess patient preferences and satisfaction. Policymakers can utilize these insights to expand the operability of OOH-PC facilities and encourage patient adherence to triage decisions, ultimately leading to a more sustainable and responsive healthcare system. The potential cost and time savings are specific to the study ED and its co-located OOH-PC. As such, our results must be interpreted in the context of institutional characteristics in combination with patient types and catchment area. However, in a fee-for-service model that does not account for severity, hence care needs, diverting low-risk patients impacts the financial balance of ED operations. Differences in healthcare infrastructure, patient demographics, and healthcare provider characteristics may limit the applicability of our results to other hospitals or regions. Further studies are needed to assess whether similar savings would be observed in different healthcare settings. Furthermore, while our analysis provides absolute estimates of potential annual savings for the study site, these figures should be interpreted in relation to the size and budget of the facility. The magnitude of cost savings will be dependent on the hospital’s total operational budget, bed capacity, patient throughput, and staffing levels. Expressing savings as a share of the total ED budget or per hospital bed would allow for more meaningful benchmarking across sites. However, such contextual budgetary data were beyond the scope of this study. Future research could incorporate these relative indicators to facilitate comparisons between ED–OOH-PC configurations of varying scale and resource intensity

Our findings for the remaining ED patients regarding the intervention’s impact on differences in costs, time spent at the facility, and hospitalization probability are inconclusive when comparing low-risk patients at the ED during the intervention to low-risk patients at the ED during the control. Similarly, the intervention’s impact on costs, time spent at the facility (excluding hospitalizations) is inconclusive when comparing high-risk patients at the ED during the intervention to high-risk patients at the ED during the control. These inconclusive findings between these subgroups could reflect established protocols and guidelines that ensure a consistent approach to diagnosing and treating patients, irrespective of the intervention. Further, the proportion of low-risk patients was relatively small, and we did not account for the total number of ED patients per weekend or the temporal patterns in patient presentations.

The current study assumes that all low-risk patients who present at the ED require out-of-hours care. Some healthcare professionals argue that certain non-urgent cases may not require out-of-hours care and could be managed within regular-hours primary care settings (Keizer *et al*., 2016). While diverting patients from the ED to OOH-PC might relieve the workload of the former, one cannot underestimate the additional efforts required from GPs (Keizer *et al*., 2016). However, based on anecdotal input from clinical experts involved in the TRIAGE trial, the risk of overcrowding at the GPC during the study period was minimal, and the contribution of triaged ED patients represented only about 7% of the total GPC workload. This indicates that the introduction of the triage-based redirection system did not substantially burden the OOH-PC service, and capacity constraints were unlikely to have influenced the observed outcomes. Outside of the trial context, similar levels of redirection would not be expected to cause overcrowding. Furthermore, having many low-risk patients at the ED can negatively impact the motivation of ED doctors, as they may feel their skills are underutilized and their time diverted from more critical cases (Kase and Doolittle, 2023). Therefore, the aim of enhancing out-of-hours continuity of care through OOH-PC requires careful balancing and consideration (Phiri *et al*., 2024).

In this study, we did not compare the aggregated patient populations at OOH-PC and ED care in terms of differences in costs, time spent at the facility, and probability of hospitalization, due to differences in patient case mix. In addition, the proportion of high-risk vs low-risk patients at the ED is impacted by the triage but has many other confounding factors. As a result, all analyses were stratified by risk level.

The gatekeeping role of GPs, requiring patients to contact OOH-PC before going to the ED, varies across countries (Smits *et al*., 2019). In Belgium, there is no mandatory gatekeeping to secondary or tertiary care, and ED care has no on-the-spot patient copayment in contrast to OOH-PC (Smits *et al*., 2019). Smits et al., found that utilization of OOH-PC in Belgium was relatively low compared to the Netherlands, where OOH-PC does play a gatekeeping role (Smits *et al*., 2019). The consequence of low-risk patients not seeking care at OOH-PC when available is that they may experience longer time at an ED potentially leading to a congestion of EDs (Caldas and Soares, 2022). Future policy decisions could consider the potential benefits of implementing a gatekeeping system for low-risk patients while also considering an efficient and well-functioning referral system for easy and prompt referral of high-risk patients.

Previous analysis of the TRIAGE trial data found that low socio-economic status was associated with increased refusal of advice to seek OOH-PC (Homburg *et al*., 2022). This suggests that the benefits of treating low-risk patients at the OOH-PC may disproportionately disfavor individuals from lower socio-economic backgrounds, which raises equity concerns. Addressing these disparities ensures that the cost-saving benefits and productivity gains of directing low-risk patients to OOH-PC are realized across all populations.

Including an established scoring system to quantify crowding in emergency departments, such as the National Emergency Department Overcrowding Score (Age UK, 2025), would have been interesting for our analysis, but this was not measured during the TRIAGE trial. As such, we are unable to include a validated crowding score. In addition, our time-to-treatment setup does not provide information on the full time spent at the facility, so an estimate of occupancy, or crowding, based on these numbers carries high uncertainty. Given both considerations, we did not include these covariates. However, future research should consider incorporating crowding indices or real-time patient flow data to better account for system-level factors that may influence treatment time and resource use.

## Limitations

The number of records missing time spent at the facility was notably high at 42%. These values remained relatively constant throughout the year, with slight increases observed in November and December. Given the large number of missing records, imputation methods would not be effective. Note that differences between clinicians are accounted for in the mixed-effects modeling.

In this study, discharge time was not available; therefore, we used the time between the recording of the nurse-led triage and the recording of the diagnosis by the clinician in the electronic patient file as a proxy for time spent at the facility. We assumed that both GPs and ED clinicians generally register the diagnosis in the electronic patient file at discharge. While the flow of patients at the OOH-PC is sequential, with the recording of the diagnosis typically coinciding with patient departure, an ED clinician usually sees patients interchangeably. In this regard, factors such as workload, clinical workflow, and individual habits could lead to delays in recording, potentially introducing measurement bias. By omitting extreme outliers and by employing mixed-effects models to account for both patient and clinician-level variability, we aimed to reduce this bias and enhance the reliability of our estimates by focusing on incremental effects. If ED clinicians systematically register the diagnosis before discharge, the current estimates underestimate the time spent at the ED, and thus the observed time gain is an underestimation. Conversely, if the registration of the diagnosis is commonly done a long time after patient discharge, we are overestimating the time spent at the facility, and therefore our observations require some reservation. However, we assume it was common practice to register the diagnosis (if this is done) when prescriptions are prepared to be provided to the patient at discharge. Future research could benefit from directly measuring time spent at the facility, where feasible, to further validate our findings.

We did not account for 3% of patients who left without being seen, while it reflects real-world patient behaviour, aligning with natural healthcare utilization patterns. As stated in previous work, the proportion of patients who left without being seen increased during intervention weekends when allocated to the OOH-PC, with anecdotal evidence that they attended their own GP afterwards (Morreel *et al*., 2021). However, patients leaving without being seen could also be seen as a marker of low risk, as these patients may have been reassured by the triage nurse or reconsidered their need for care, something that is easier to do while in transit than when waiting in a clinic (Kennedy *et al*., 2008; Li *et al*., 2019). In our study, leaving without being seen impact on time and cost is theoretically bi-directional, with shorter queues versus removal of very low-cost/short-visit cases (Kennedy *et al*., 2008; Li *et al*., 2019). However, given the small rate of leaving without being seen (3%), any bias is likely limited.

Immediate short-term costs and effects are a relevant initial step to inform policy decisions. However, this study has several limitations pertaining to the full range of costs and effects. Firstly, it lacks longitudinal data to investigate patient follow-up costs and effects. OOH-PC and ED care may necessitate different follow-up visits, potentially accumulating additional costs and effects. Secondly, this study only considered direct medical costs from the patient and healthcare payer perspective, excluding nonmedical costs such as caregiver and childcare costs that impact society. Lastly, the ambulatory costs for hospitalized patients did not reflect the total costs incurred at the ED by these patients, as the excluded admission related costs, underestimating the full range of costs.

When estimating potential annual cost and time savings, our analysis assumes a constant patient flow when scaling from the 37-weekend intervention period to a 52-weekend, full-year period. This assumption may not fully capture real-world variability in patient volumes, as the 37 weekends from 1^st^ March to 31^st^ December, only partially cover the months with the usual surge in high healthcare use. Seasonal trends such as influenza outbreaks, holiday periods, and weather-related health issues impact patient volumes. Future research should incorporate seasonal adjustments to enhance the accuracy of long-term projections. Another limitation of this analysis is its focus solely on weekends, excluding weekday out-of-hours periods. Consequently, the results do not represent the broader range of patient types and health issues encountered out-of-hours throughout the entire week.

## Generalizability of findings

While we demonstrated the relative advantages of OOH-PC for low-risk patients and contend that our findings may apply to similar settings, it is imperative to acknowledge that the medical costs encompassed in our analysis build upon the Belgian healthcare system. Furthermore, the nature of collaboration between OOH-PC services and EDs can vary; these two may be integrated or co-located, as observed in our study, while in other settings, they may operate separately. This organizational separation is observed at other sites in Belgium as well as in High-Risk Care Centers in Canada (Berchet, 2016). Diversity also exists in the types of healthcare professionals delivering OOH-PC across different countries. In our study, OOH-PC is delivered by GPs, whereas countries such as Denmark and the UK also incorporate nurse-led OOH-PC services (Steeman *et al*., 2020). Such organizational and operational diversities influence the costs and utilization patterns associated with OOH-PC.

The transferability of our findings to for example the United Kingdom (UK) context warrants cautious interpretation. Unlike Belgium’s mixed funding model where OOH-PC involves point-of-care payment and ED billing occurs post-visit, patients in the UK’s National Health Service (NHS) do not pay out-of-pocket fees in either urgent primary care or ED settings (Age UK, 2025). Further, nurse practitioners play a greater role in managing low-acuity cases in both ED and primary care settings in the UK compared with Belgium (Steeman *et al*., 2020).These factors likely impact the effect of diverting low-risk patients in the UK compared to our setting. However, ED crowding remains a persistent challenge in the UK (Boyle *et al*., 2021), and several NHS strategies emphasize the need to redirect avoidable demand toward primary care, suggesting that the core mechanism observed in our study may still be relevant even though the magnitude of economic gain differs.

To enhance the generalizability of the findings of this study to other settings, an “ingredients approach” to costing may be employed, with unit costs averaged across various settings (Špacírová *et al*., 2020). However, we are convinced that several similarities can be drawn with other healthcare systems, and therefore our conclusions may be adopted more broadly.

## Conclusions and further research

This Belgian study demonstrates that managing low-risk patients at an OOH-PC facility instead of at an ED constituted a cost- and time-saving strategy. Specifically, OOH-PC reduced the estimated time at the facility by approximately 1 hour when compared to low-risk patients at the ED. Additionally, these outcomes are achieved with an average cost saving of €24 per patient, thereby highlighting the economic value of OOH-PC. Moreover, managing low-risk patients at the OOH-PC can bring about health system cost savings for the study ED and OOH-PC, ranging from €20,000 to €50,000 annually, depending on the assumed number of eligible low-risk patients presenting at the ED. These findings highlight the potential for OOH-PC to enhance healthcare delivery by more efficiently addressing low-risk patients and providing a scalable model for cost-effective care. These improved outcomes support Sustainable Development Goal number 3, which aims to ensure healthy lives and promote well-being for all at all ages (United Nations, (2023)). Further, given that only 10% of patients were diverted to OOH-PC in this study, future research should examine whether additional diversion leads to increased waiting times for low-risk patients with limited GP capacity. Understanding the extent of this impact is crucial for optimizing patient flow and resource allocation in healthcare settings.

While using the time from the nurse-led triage to the registration of a diagnosis by a clinician as a proxy for time spent at the facility provides a practical estimate, future research that directly measures time spent at the facility will enhance the robustness of our conclusions. Additionally, future research should prioritize the evaluation of long-term outcomes and broader patient-centered and health system metrics, such as continuity of care, the uptake of health-promotive and preventative healthcare services (Phiri *et al*., 2024), and patient satisfaction. While our findings are informative for policy decisions about short-term impacts, hospitalization during the visit, and time spent at the facility, further research is required to consider multifaceted effects to foster a better understanding of the impact of the intervention to inform policy decisions. The impact of the intervention on high-risk patients remains inconclusive, but this is crucial information for evaluating the intervention’s impact on the efficiency of ED care. Additionally, exploring other data sources to incorporate more variables can better account for potential confounders. Furthermore, incorporating seasonal adjustments to the annual costs and effects savings would enhance the accuracy of long-term projections.

## Supporting information

10.1017/S146342362610142X.sm001 Phiri et al. supplementary material Phiri et al. supplementary material

## Supplementary material

The supplementary material for this article can be found at https://doi.org/10.1017/S146342362610142X.

## Figure 1. Long description

A flowchart illustrating the triage process for assigning patients to either the Emergency Department (ED) or Out-of-Hours Primary Care (OOH-PC). The flowchart starts with five categories: MTS Category 1 (Immediate), MTS Category 2 (Very Urgent), MTS Category 3 (Urgent), MTS Category 4 (Standard), and MTS Category 5 (Non-urgent). MTS Categories 1, 2, and 3 converge into a single path leading to eMTS Assignment: ED. MTS Categories 4 and 5 converge into another path. This path includes a decision point labeled Additional Distriminators. If the answer is yes, it leads to eMTS Assignment: ED. If the answer is no, it proceeds to another decision point labeled Expected need for hospital care. If the answer is yes, it leads to eMTS Assignment: ED. If the answer is no, it leads to eMTS Assignment: OOH-PC.

Navigate back to Figure 1..

## Table 1. Long description

A table with two columns and six rows. The first column is labeled ‘Cost category’ and the second column is labeled ‘Description’. Row 1: Ambulatory physician fees, no description. Row 2: Medical imaging, E.g. radiology procedures. Row 3: Technical procedures, E.g. sutures, plastering. Row 4: Medications, Medication costs only cover the medication administered to the patient during a consultation, excluding any prescriptions provided. Row 5: Non-refundable items, The non-refundable items category includes various articles requested by the patient (e.g., a toothbrush) or necessary for their medical care (e.g., crutches) (Morreel et al., 2022a). Row 6: Supplemental fees, Night-time and weekend consultation surcharges.

Navigate back to Table 1..

## Table 2. Long description

The table presents data on the number of weekends, patient population sizes, medical costs, time spent at care facilities, and hospitalization probabilities for different groups of patients. It includes five rows of data and six columns of headers. The columns are labeled as follows: Number of weekends, Population size (cost and hosp. analysis), Population size (time analysis), Medical cost (€), Time spent at care facility (minutes), and Hospitalization probability. The rows are labeled as follows: Low-risk patients at OOH-PC during the intervention, Low-risk patients at ED during the intervention, High-risk patients at ED during the intervention, Low-risk patients at ED during the control, and High-risk patients at ED during the control. Each row provides specific values for each column, including mean values and bias-corrected and accelerated 95% confidence intervals where applicable. Notable trends include variations in medical costs, time spent at care facilities, and hospitalization probabilities across different patient groups and conditions.

Navigate back to Table 2..

## Table 3. Long description

The table compares cost-effectiveness results for time spent at the facility and hospitalization across different patient populations. It has four rows and eight columns. The columns are labeled as Comparisons, Incremental cost (B C a 95 percent C I), Reduced time at facility (B C a 95 percent C I) (min), I C E R – cost per reduction in time spent at the facility (€/min) (€/hour), I N M B (time spent at the facility) (95 percent C I), I N M B – percent positive, Reduced Hospitalization probability (B C a 95 percent C I), and I C E R – Hospitalization prob. The rows are labeled as (A) Low-risk patients at O O H-P C during the intervention vs low-risk patients at the ED during the intervention, (B) Low-risk patients at O O H-P C during the intervention vs low-risk patients at ED during the control, (C) Low-risk patients at ED during the intervention vs low-risk patients at ED during the control, and (D) High-risk patients at ED during the intervention vs high-risk patients at ED during the control. Each row provides specific values for the columns.

Navigate back to Table 3..

## References

[ref1] Age UK (2025) NHS services: Factsheet 44 [Internet]. Available at https://www.ageuk.org.uk/siteassets/documents/factsheets/fs44_nhs_services_fcs.pdf?utm_source=chatgpt.com (cited 2025 Nov 4).

[ref2] Ben-Yakov M , Kapral MK , Fang J , Li S , Vermeulen MJ and Schull MJ (2015) The association between emergency department crowding and the disposition of patients with transient ischemic attack or minor stroke. Academic Emergency Medicine 22(10), 1145–1154. 10.1111/ACEM.12766 26398233

[ref3] Berchet C (2015) Emergency Care Services: Trends, Drivers and Interventions to Manage the Demand. OECD Health Working Papers. 83. 10.1787/5jrts344crns-en

[ref4] Berchet C (2016) The organisation of out-of-hours primary care in OECD countries. OECD Health Working Papers. 89. Available at 10.1787/5jlr3czbqw23-en

[ref5] Boyle A , Higginson I , Sarsfield K and Kumari P (2021) RCEM Acute Insight Series: Crowding and Its Consequences [Internet]. Available at https://www.rcem.ac.uk/RCEM/Quality_Policy/Policy/RCEM_CARES/RCEM/Quality- (cited 2025 Oct 27).

[ref6] Broekman S , Van Gils-Van Rooij E , Meijboom B , De Bakker D and Yzermans C (2017) Do out-of-hours general practitioner services and emergency departments cost more by collaborating or by working separately? A cost analysis. Journal of Primary Health Care 9(3), 212–219. 10.1071/HC17015 29530174

[ref7] Caldas FM and Soares C (2022) A Temporal Fusion Transformer for Long-term Explainable Prediction of Emergency Department Overcrowding. Communications in Computer and Information Science. Available at 1752 CCIS: 71–88. 10.1007/978-3-031-23618-1_5

[ref8] Chesteen SA , Warren SE and Woolley FR (1986) A comparison of family practice clinics and free-standing emergency centers: Organizational characteristics, process of care, and patient satisfaction. The Journal of Family Practice 23(4), 377–382. 3760804

[ref9] Colliers A , Bartholomeeusen S , Remmen R , Coenen S , Michiels B , Bastiaens H , Royen PV , Verhoeven V , Holmgren P , De Ruyck B and Phillips H (2016) Improving Care and Research Electronic Data Trust Antwerp (iCAREdata): A research database of linked data on out-of-hours primary care. BMC Research Notes 9(1), 1–7. 10.1186/S13104-016-2055-X/FIGURES/1 27142361 PMC4855754

[ref10] Cooper A , Davies F , Edwards M , Anderson P , Carson-Stevens A , Cooke MW , Donaldson L , DALE J , Evans BA , Hibbert PD , Hughes TC , Porter A , Rainer T , Siriwardena A , Snooks H and Edwards A (2019) The impact of general practitioners working in or alongside emergency departments: A rapid realist review. BMJ Open 9(4), e024501. 10.1136/BMJOPEN-2018-024501 PMC6500276 30975667

[ref11] Durand AC , Palazzolo S , Tanti-Hardouin N , Gerbeaux P , Sambuc R and Gentile S (2012) Nonurgent patients in emergency departments: Rational or irresponsible consumers? Perceptions of professionals and patients. BMC Research Notes 5(1), 1–9. 10.1186/1756-0500-5-525/TABLES/3 23006316 PMC3515357

[ref12] Elkum N , Fahim M , Shoukri M and Al-Madouj A (2009) Which patients wait longer to be seen and when? A waiting time study in the emergency department. Eastern Mediterranean Health Journal 15(2), 416–424. 10.26719/2009.15.2.416 19554989

[ref13] Flaherty KE , Klarman MB , Cajusma Y , Schon J , Exantus L , Beau de Rochars VM , Baril C , Becker TK , Nelson EJ (2022) A nighttime telemedicine and medication delivery service to avert pediatric emergencies in Haiti: An exploratory cost-effectiveness analysis. American Journal of Tropical Medicine and Hygiene 106(4), 1063. 10.4269/AJTMH.21-1068 35189597 PMC8991343

[ref14] Francetic I , Meacock R and Sutton M (2024) Free-for-all: Does crowding impact outcomes because hospital emergency departments do not prioritise effectively? Journal of Health Economics 95, 102881. 10.1016/J.JHEALECO.2024.102881 38626590

[ref16] Gaughan J , Liu D , Gutacker N , Bloor K , Doran T and Benger JR (2022) Does the presence of general practitioners in emergency departments affect quality and safety in English NHS hospitals? A retrospective observational study. BMJ Open 12, 55976. 10.1136/bmjopen-2021-055976 PMC8867306 35197350

[ref45] Goldstein H and Healy MJR (1995) The Graphical Presentation of a Collection of Means. Journal of the Royal Statistical Society 158(1), 175–177. 10.2307/2983411

[ref17] Homburg I , Morreel S , Verhoeven V , Monsieurs KG , Meysman J , Philips H and De Graeve D (2022) Non-compliance with a nurse’s advice to visit the primary care provider: An exploratory secondary analysis of the TRIAGE-trial. BMC Health Services Research 22(1), 1–11. 10.1186/S12913-022-07904-8/FIGURES/2 35395840 PMC8994354

[ref18] Kase J and Doolittle B (2023) Job and life satisfaction among emergency physicians: A qualitative study. PLoS One 18(2) e0279425. 10.1371/JOURNAL.PONE.0279425 36827313 PMC9955602

[ref19] Keizer E , Maassen I , Smits M , Wensing M and Giesen P (2016) Reducing the use of out-of-hours primary care services: A survey among Dutch general practitioners. European Journal of General Practice 22(3), 189–195. 10.1080/13814788.2016.1178718 27248713

[ref20] Kennedy M , MacBean CE , Brand C , Sundararajan V and McD Taylor D (2008) Review article: Leaving the emergency department without being seen. Emergency Medicine Australasia 20(4), 306–313. 10.1111/J.1742-6723.2008.01103.X 18782204

[ref21] Li DR , Brennan JJ , Kreshak AA , Castillo EM and Vilke GM (2019) Patients who leave the emergency department without being seen and their follow-up behavior: A retrospective descriptive analysis. The Journal of Emergency Medicine 57(1), 106–113. 10.1016/J.JEMERMED.2019.03.051 31078346

[ref22] Martins HMG , De Castro Dominguez Cuña LM and Freitas P (2009) Is Manchester (MTS) more than a triage system? A study of its association with mortality and admission to a large Portuguese hospital. Emergency Medicine Journal 26(3), 183–186. 10.1136/EMJ.2008.060780 19234008

[ref27] Møller AD , Christiansen DH , Bell C , Fredberg U and Vedsted P (2018) 24-hour access outpatient clinic for patients with exacerbation of chronic disease: A before-after cohort study of differences in acute healthcare utilisation. BMC Health Services Research 18(1), 1–9. 10.1186/S12913-018-3475-1/TABLES/2 30153833 PMC6114062

[ref23] Moore S , Young T , Irving A , Goodacre S , Brennan A and Amos Y (2021) Controlled observational study and economic evaluation of the effect of city-centre night-time alcohol intoxication management services on the emergency care system compared with usual care. Emergency Medicine Journal 38(7), 504–510. 10.1136/EMERMED-2019-209273 33148772

[ref24] Morreel S , Homburg I , Philips H , De Graeve D , Monsieurs KG , Meysman J , Lefevere E and Verhoeven V (2022a) Cost effects of nurse led triage at an emergency department with the advice to consult the adjacent general practice cooperative for low-risk patients, a cluster randomised trial. Health Policy 126(10), 980–987. 10.1016/J.HEALTHPOL.2022.08.002 35963797

[ref25] Morreel S , Philips H , de Graeve D , Monsieurs KG , Kampen JK , Meysman J , Lefevere E and Verhoeven V (2021) Triaging and referring in adjacent general and emergency departments (the TRIAGE trial): A cluster randomised controlled trial. PLoS One 16(11), e0258561. 10.1371/JOURNAL.PONE.0258561 34731198 PMC8565772

[ref26] Morreel S , Verhoeven V , Philips H , Meysman J , Homburg I , De Graeve D and Monsieurs KG (2022b) Differences in emergency nurse triage between a simulated setting and the real world, post hoc analysis of a cluster randomised trial. BMJ Open 12(7), e059173. 10.1136/BMJOPEN-2021-059173 PMC9252194 35777880

[ref28] Phiri J , Morreel S , De Graeve D , Philips H , Beutels P , Verhoeven V and Willem L (2024) The need for a broad perspective when assessing value-for-money for out-of-hours primary care. Primary Health Care Research & Development 25, e37. 10.1017/S1463423624000318 39301601 PMC11464846

[ref29] Poole SR , Schmitt BD , Carruth T , Peterson-Smith A and Slusarski M (1993) After-hours telephone coverage: The application of an area-wide telephone triage and advice system for pediatric practices. Pediatrics 92(5), 670–679. 8414853

[ref30] Roukema J , Steyerberg EW , van Meurs A , Ruige M , van der Lei J and Moll HA (2006) Validity of the Manchester Triage System in paediatric emergency care. Emergency Medicine Journal 23(12), 906–910. 10.1136/EMJ.2006.038877 17130595 PMC2564249

[ref31] Schoenmakers B , Van Criekinge J , Boeve T , Wilms J , Van Der Mullen C and Sabbe M (2021) Co-location of out of hours primary care and emergency department in Belgium: Patients’ and physicians’ view. BMC Health Services Research 21(1), 282. 10.1186/S12913-021-06281-Y 33771152 PMC7995743

[ref32] Smits M , Colliers A , Jansen T , Remmen R , Bartholomeeusen S and Verheij R (2019) Examining differences in out-of-hours primary care use in Belgium and the Netherlands: A cross-sectional study. European Journal of Public Health 29(6), 1018–1024. 10.1093/EURPUB/CKZ083 31086964 PMC6896980

[ref44] Špacírová Z , Epstein D , García-Mochón L , Rovira J , de Labry Lima AO and Espín J (2020) A general framework for classifying costing methods for economic evaluation of health care. European Journal of Health Economics 21(4), 529–542. 10.1007/S10198-019-01157-9/TABLES/5 PMC8149350 31960181

[ref33] STATBEL (2021) Average gross income: 3,758 euros per month [Internet]. Available at https://statbel.fgov.be/en/news/average-gross-income-3758-euros-month (cited 2024 Nov 26).

[ref34] Steeman L , Uijen M , Plat E , Huibers L , Smits M and Giesen P (2020) Out-of-hours primary care in 26 European countries: An overview of organizational models. Family Practice 37(6), 744–750. 10.1093/FAMPRA/CMAA064 32597962 PMC7699311

[ref35] Sterner SE , Coco T , Monroe KW , King WD and Losek JD (2012) A new after-hours clinic model provides cost-saving, faster care compared with a pediatric emergency department. Pediatric Emergency Care 28(11), 1162–1165. 10.1097/PEC.0B013E318271733E 23114241

[ref36] Stinnett AA and Paltiel AD (1997) Estimating CE Ratios under Second-order Uncertainty: The mean ratio versus the ratio of means. Medical Decision Making 17(4), 483–489. 10.1177/0272989X9701700414 9343807

[ref37] United Nations (2023) Global Sustainable Development Report 2023: Key Messages. Available at: https://sdgs.un.org/goals (cited 2025 Apr 1).

[ref38] Van Der Wulp I , Schrijvers AJP and Van Stel HF (2009) Predicting admission and mortality with the Emergency Severity Index and the Manchester Triage System: A retrospective observational study. Emergency Medicine Journal 26(7), 506–509. 10.1136/EMJ.2008.063768 19546272

[ref39] Walle Z , Worku F , Sraneh Y , Melese D , Fufa T , Yesuf EA and Berihun G (2024) Overall time spent by clients from entry to exit and associated factors in out-patient departments in public hospitals of Jimma Zone southwest, Ethiopia. PLoS One 19(3), e0296630. 10.1371/JOURNAL.PONE.0296630 38451898 PMC10919670

[ref40] Weichle T , Hynes DM , Durazo-Arvizu R , Tarlov E and Zhang Q (2013) Impact of alternative approaches to assess outlying and influential observations on health care costs. SpringerPlus 2(1), 1–11. 10.1186/2193-1801-2-614 24303338 PMC3843184

[ref41] Wicklin R (2017) The DO Loop [Internet]. The bias-corrected and accelerated (BCa) bootstrap interval. Available at https://blogs.sas.com/content/iml/2017/07/12/bootstrap-bca-interval.html (cited 2025 Apr 1)

[ref42] York Health Economics Consortium (2025) Net Monetary Benefit [Internet]. Available at https://www.yhec.co.uk/glossary-term/net-monetary-benefit/ (cited 2026 Mar 23).

[ref43] Zhang L , Wang B and Fu C (2025) The effect of patient participation on trust in primary health care physicians among patients with chronic diseases: The mediating role of perceived value. Front Public Health 13, 1586123. 10.3389/FPUBH.2025.1586123/BIBTEX 40438066 PMC12116563

