# Supplementary material for: Economic evaluation of diverting low-risk patients from the emergency department to adjacent out-of-hours primary care in Belgium
Source: Prim Health Care Res Dev. 2026 Jul 30;27:e81. doi: 10.1017/S146342362610142X (PMC13439030; doi:10.1017/S146342362610142X)
Supplement: Supplementary file 1 [file S146342362610142Xsup001.pdf]

APPENDIX A

Description of the Belgian healthcare system

In Belgium, formal organisation of OOH-PC by general practitioner cooperatives (GPCs) began in 2003 (Schoenmakers *et al.*, 2021). Since then, OOH-PC facilities are either integrated with EDs to streamline patient flow or operate independently (Schoenmakers *et al.*, 2021). The healthcare system in Belgium is based on the principles of independent medical practice, direct access (without mandatory gatekeeping), and the freedom of choice of physician and healthcare facility (Gerkens and Merkur, 2024). Therefore, both OOH-PCs and EDs are accessible at the patients' discretion. The Belgian healthcare system operates under a compulsory social health insurance model, funded through social security contributions and taxation (Gerkens and Merkur, 2024). The "National Institute for Health and Disability Insurance" reimburses medical costs based on predefined tariffs while patients contribute with co-payments, which may be partially covered by supplemental private insurance (Gerkens and Merkur, 2024). Both OOH-PC and ED services in Belgium follow a fee-for-service model, but co-payment mechanisms differ. At OOH-PC, patients pay upfront, with the insurance fund reimbursing a portion of the cost while at EDs, patients do not pay upfront but instead receive a bill afterwards, postponing the financial burden for patients (Morreel, Homburg, *et al.*, 2022). Additionally, patients attending either OOH-PC or ED outside of regular working hours may incur emergency supplemental surcharges, increasing costs compared to regular-hour care (Morreel, Homburg, *et al.*, 2022).

APPENDIX B

Model Building and Selection Procedure

To identify the most appropriate regression models for the cost and effect analyses, eight candidate model specifications were evaluated for each outcome, differing in their combination of fixed and random effects (Table B1). Both forward and backward stepwise elimination procedures were applied to guide model selection.

Clinician ID was included as a random effect in all models to account for between-clinician variation. The covariates age and sex were alternately specified as either fixed or random effects, depending on the model structure. Risk-level, Setting, and Intervention arm were included as fixed effects in all candidate models. In half of the specifications, all plausible interaction terms among these variables (Risk-level × Setting × Intervention arm) were also included to test their contribution to model fit.

For each iterative forward and backward elimination process, the model with the lowest Akaike Information Criterion (AIC) value was selected as providing the best balance between model fit and simplicity. The covariates retained in the best-fitting model from each specification, together with the corresponding AIC values for the cost, time at facility, and hospitalisation outcomes, are summarised in Table B1. The model yielding the lowest AIC across all eight specifications was selected as the final model for each outcome analysis.

For costs, the model retaining the three-way interaction between Risk-level × Setting × Intervention arm with Clinician ID as a random effect (and age and gender included as fixed covariates) achieved the lowest AIC (AIC = 91 436.67) and was selected for the main analysis. For time spent at the facility, the model retaining the same three-way interaction with Clinician ID as a random effect and age and gender as fixed effects yielded the lowest AIC (AIC = 47 474.10). For hospitalisation, the

model retaining the three-way interaction with Clinician ID and age as random effects provided the lowest AIC (AIC = 5 496.78) and was selected for analysis.

Table B1 Model specifications, best-fitting models, and corresponding Akaike Information Criterion (AIC) values from forward and backward stepwise elimination procedures

	Model specification	Cost: Best Model		Time at facility: Best Model		Hospitalization: Best Model	
		**Variables in best model**	**AIC**	**Variables in best model**	**AIC**	**Variables in best model**	**AIC**
1	**Fixed effects:** Risk-level, Setting, Intervention arm, Risk-level*Setting*Intervention arm<br><br>**Random effect:** Clinician, age and gender	**Fixed effects:** Risk-level, Setting, Intervention arm, Intervention arm*Setting, Setting*Risk-level,<br><br>**Random effect:** Clinician, age and gender	91538.13	**Fixed effects:** Risk-level*Setting*Intervention arm<br><br>**Random effect:** Clinician, age and gender	47527.29	**Fixed effects:** Risk-level, Setting, Intervention arm, Intervention arm*Setting, Setting*Risk-level,<br><br>**Random effect:** Clinician, age and gender	5498.8
2	**Fixed effects:** Risk-level, Setting, Intervention arm<br><br>**Random effect:** Clinician, age and gender	**Fixed effects:** Risk-level, Setting, Intervention arm<br><br>**Random effect:** Clinician, age and gender	91545.08	**Fixed effects:** Setting<br><br>**Random effect:** Clinician, age and gender	47538.86	**Fixed effects:** Risk-level, Setting, Intervention arm<br><br>**Random effect:** Clinician, age and gender	5496.8
3	**Fixed effects:** Risk-level, Setting, Intervention arm, Risk-level*Setting*Intervention arm, age, gender<br><br>**Random effect:** Clinician	**Fixed effects:** Risk-level*Setting*Intervention arm, age, gender<br><br>**Random effect:** Clinician	91436.67	**Fixed effects:** Risk-level*Setting*Intervention arm, age, gender<br><br>**Random effect:** Clinician	47474.10	**Fixed effects:** Risk-level, Setting, Intervention arm, age<br><br>**Random effect:** Clinician	5606.136
4	**Fixed effects:** Risk-level, Setting, Intervention arm, age, gender<br><br>**Random effect:** Clinician	**Fixed effects:** Risk-level, Setting, Intervention arm, age, gender<br><br>**Random effect:** Clinician	91441.26	**Fixed effects:** Risk-level, Setting, Intervention arm, age, gender<br><br>**Random effect:** Clinician	47478.48	**Fixed effects:** Risk-level, Setting, Intervention arm, age<br><br>**Random effect:** Clinician	5606.136

5	**Fixed effects:** Risk-level, Setting, Intervention arm, Risk-level*Setting*Intervention arm, age<br><br>**Random effect:** Clinician, gender	**Fixed effects:** Risk-level*Setting*Intervention arm, age<br><br>**Random effect:** Clinician, gender	91443.69	**Fixed effects:** Risk-level*Setting*Intervention arm, age<br><br>**Random effect:** Clinician, gender	47480.36	**Fixed effects:** Risk-level*Setting*Intervention arm, age<br><br>**Random effect:** Clinician, gender	5608.2
6	**Fixed effects:** Risk-level, Setting, Intervention arm, age<br><br>**Random effect:** Clinician, gender	**Fixed effects:** Risk-level, Setting, Intervention arm, age<br><br>**Random effect:** Clinician, gender	91448.28	**Fixed effects:** Risk-level, Setting, Intervention arm, age<br><br>**Random effect:** Clinician, gender	47484.7	**Fixed effects:** Risk-level, Setting, Intervention arm, age<br><br>**Random effect:** Clinician, gender	5608.2
7	**Fixed effects:** Risk-level, Setting, Intervention arm, Risk-level*Setting*Intervention arm, gender<br><br>**Random effect:** Clinician, age	**Fixed effects:** Risk-level*Setting*Intervention arm, gender<br><br>**Random effect:** Clinician, age	91531.20	**Fixed effects:** Risk-level*Setting*Intervention arm, gender<br><br>**Random effect:** Clinician, age	47520.83	**Fixed effects:** Risk-level*Setting*Intervention arm<br><br>**Random effect:** Clinician, age	5496.78
8	**Fixed effects:** Risk-level, Setting, Intervention arm, gender<br><br>**Random effect:** Clinician, age	**Fixed effects:** Risk-level, Setting, Gender<br><br>**Random effect:** Clinician, age	91538.16	**Fixed effects:** Risk-level, Setting, Intervention arm, gender<br><br>**Random effect:** Clinician, age	47525.19	**Fixed effects:** Risk-level*Setting*Intervention arm<br><br>**Random effect:** Clinician, age	5496.8

APPENDIX C

Data analysis

We conducted all analyses using R (version 4.4.0 - 2024-04-24) within the RStudio environment (version 2023.09.1+494). For the numerical cost and time spent at the facility models section, the lmerTest package (version 3.1-3) was used for the linear and mixed-effects model, while for the binary hospitalisation variable, the MuMIn package (version 1.48.4) was used for the generalised linear mixed-effects model. Both are included in the forward and backward stepwise model selection processes. To select the best models, the function AIC() was used, which is part of the base R. To perform regression analyses, both mixed-effects and generalised linear mixed-effect models were adopted from the lme4 package (version 1.1-35.3). Thereafter, the emmeans package (version 1.10.4) was used to predict means from the regressions. The function rgamma, which was used for the PSA, was also part of the base R.

APPENDIX D

The stepwise creation of the analytical datasets

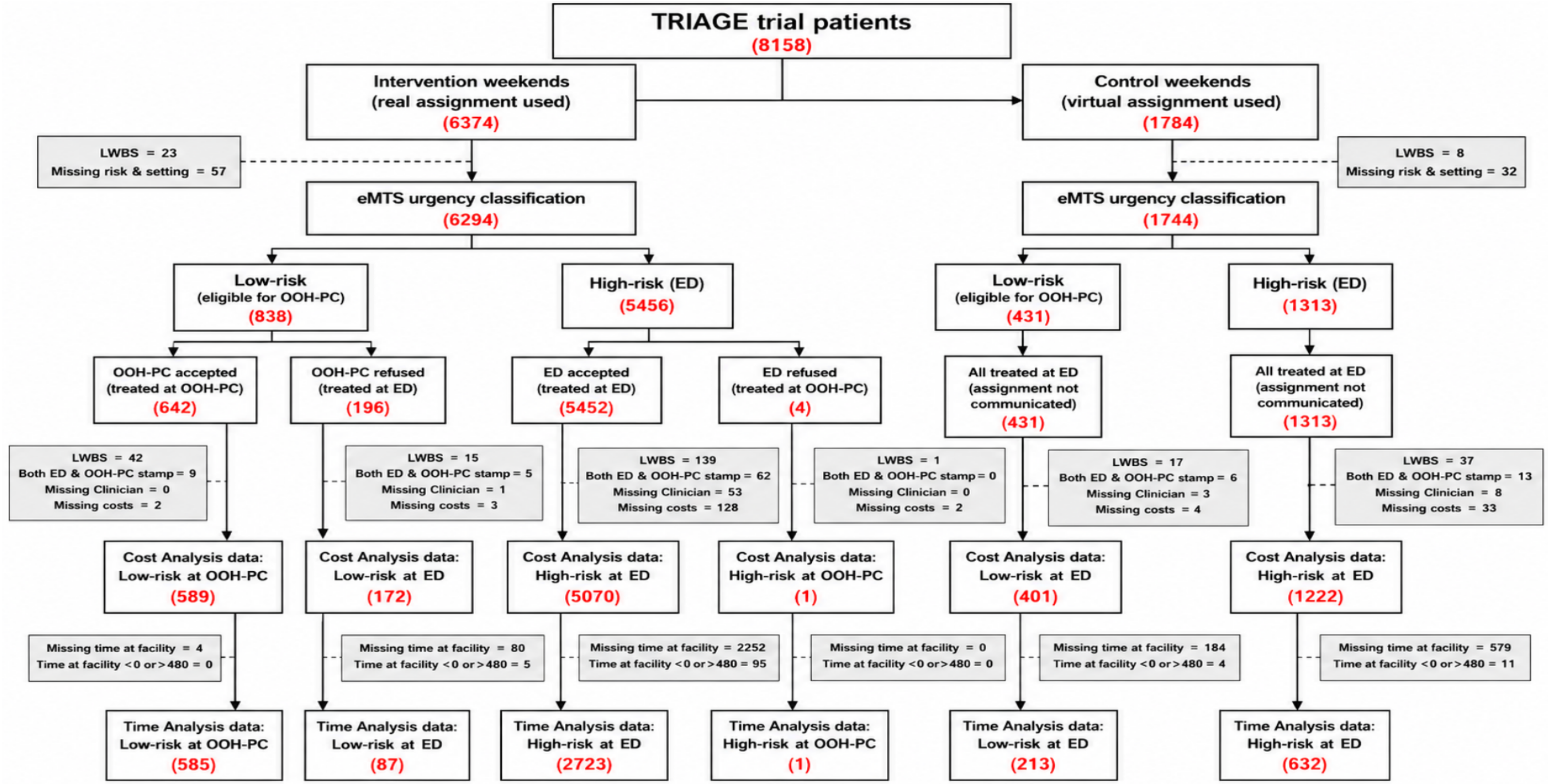

[1]

Legend: ED- Emergency Department; eMTS- extended Manchester Triage System; OOH-PC - Out-of-Hours Primary Care; LWBS- Left Without Being Seen.

[1] The "High-risk at OOH-PC (n = 1)" arm falls outside the five primary analysis groups and is therefore excluded from the totals cited in the main text

APPENDIX E

Sensitivity analysis with different WTP to calculate the INMB

Comparisons	WTP = 0.0904 euro/min		WTP = 0.2893 euro/min		WTP = 0.4339 euro/min		WTP = 0.6328 euro/min		WTP = 0.7232 euro/min		WTP = 1.0848 euro/min	
	INMB (time spent at the facility (95% CI))	INMB - % positive	INMB (time spent at the facility (95% CI))	INMB - % positive)	INMB (time spent at the facility (95% CI))	INMB - % positive)	INMB (time spent at the facility (95% CI))	INMB - % positive	INMB (time spent at the facility (95% CI))	INMB - % positive	INMB (time spent at the facility (95% CI))	INMB - % positive
(A) Low-risk patients at OOH-PC during the intervention vs low-risk patients at the ED during the intervention	30.3 (5.6, 55.1)	83.2%	44.4 (17.7, 71)	85.0%	54.5 (25.4, 83.6)	85.9%	68.5 (34.9, 102.1)	86.9%	74.9 (39.0, 110.8)	87.4%	100.4 (54.1, 146.7)	88.6%
(B) Low-risk patients at OOH-PC during the intervention vs low-risk patients at ED during the control	29.3 (8.1, 50.6)	82.3%	42.1 (18.8, 65.3)	84.1%	51.3 (25.5, 77.1)	85.0%	64.0 (33.7, 94.4)	85.9%	69.8 (37.2, 102.5)	86.2%	92.9 (49.9, 135.9)	87.4%
(C) Low-risk patients at ED during the intervention vs low-risk patients at ED during the control	-1.0 (-21.0, 19.0)	47.6%	-2.3 (-22.8, 18.2)	46.8%	-3.2 (-24.4, 17.9)	46.4%	-4.5 (-26.9, 17.9)	45.6%	-5.1 (-28.3, 18.1)	45.3%	-7.4 (-34.1, 19.2)	44.7%
(D) High-risk patients at ED during the intervention vs high-risk patients at ED during the control	-1.0 (-8.1, 6.1)	56.7%	-1.5 (-8.8, 5.7)	57.4%	-1.9 (-9.4, 5.6)	57.8%	-2.4 (-10.3, 5.5)	58.4%	-2.6 (-10.8, 5.5)	58.9%	-3.6 (-12.9, 5.8)	59.8%
